# *Tropheryma whipplei* Detection by Nanopore Sequencing in Patients With Interstitial Lung Disease

**DOI:** 10.3389/fmicb.2021.760696

**Published:** 2021-11-29

**Authors:** Yifan Guo, Lijuan Li, Zhenzhong Li, Lingxiao Sun, Hui Wang

**Affiliations:** ^1^Department of Clinical Laboratory, Peking University People’s Hospital, Beijing, China; ^2^Institute of Medical Technology, Peking University Health Science Center, Beijing, China; ^3^Department of Pulmonary and Critical Care Medicine, National Center for Clinical Research on Respiratory Diseases, China-Japan Friendship Hospital, Beijing, China; ^4^Department of Pulmonary and Critical Care Medicine, China-Japan Friendship Hospital, Beijing, China; ^5^State Key Laboratory of Translational Medicine and Innovative Drug Development, Jiangsu Simcere Diagnostics Co., Ltd., Nanjing, China; ^6^Nanjing Simcere Medical Laboratory Science Co., Ltd., Nanjing, China

**Keywords:** *Tropheryma whipplei*, Nanopore, metagenomic next-generation sequencing, pneumonia, infection

## Abstract

*Tropheryma whipplei* is a bacterium associated with Whipple’s disease, which commonly manifests as weight loss, arthralgia, and diarrhea. The most frequently involved organs comprise the heart and eyes, in addition to the central nervous system. Few studies have explored the relationship between *T. whipplei* and pneumonia. Herein, we report three patients with interstitial lung disease (ILD) of unknown cause, whose bronchoalveolar lavage fluid (BALF) were evaluated via Nanopore sequencing. In our in-house BALF Nanopore platform, human DNA was removed with saponin, to improve the reads ratio of microorganisms/host. *T. whipplei* was the sole or most abundant pathogen in all the patients, comprising 1,385, 826, and 285 reads. The positive result was confirmed via quantitative polymerase chain reaction (PCR) with two pairs of primers (cycle threshold value: 33.26/36.29; 31.68/32.01; 28.82/28.80) and Sanger sequencing. To our knowledge, this is the first report of *T. whipplei* detection using Nanopore-based sequencing. The turnaround time was approximately 6–8 h in clinical laboratories, including less than 1 h for analysis. In conclusion, the results of this study confirm that Nanopore sequencing can rapidly detect rare pathogens, to improve clinical diagnosis. In addition, diagnosis of Whipple’s disease should be combined other laboratory findings, such as periodic acid-Schiff (PAS) staining, and considered a possibility in middle-aged men presenting with ILD and a clinical history of unexplained arthralgia and/or fever.

## Introduction

Whipple’s disease (WD) is a rare chronic disease, first described by George H. Whipple in 1907, that is caused by the bacterium *Tropheryma whipplei* (Fenollar et al., 2007). The classic Whipple’s disease manifests mainly as arthralgia, diarrhea, and weight loss and is diagnosed by the histology of small bowel biopsies (Dutly and Altwegg, 2001; Lepidi et al., 2003; Lagier et al., 2010; Fenollar et al., 2014). Since the first isolation of this bacteria (Fenollar et al., 2007), the complete genome sequence has been obtained and provides a rational choice of DNA targets for polymerase chain reaction (PCR) assays (Bentley et al., 2003; Raoult et al., 2003). Acute infections can include endocarditis (Fenollar et al., 2013), gastroenteritis (Raoult et al., 2010), bacteremia (Fenollar et al., 2010), and pneumonia (Harris et al., 2007).

Depending on the organs involved, the clinical manifestations of WD are diverse and non-characteristic. The lack of understanding of WD has significantly contributed to the disease being often misdiagnosed or undiagnosed. Since next-generation sequencing (NGS) technology was developed, metagenomic NGS (mNGS) has begun to improve the etiological diagnosis of infectious diseases (Blauwkamp et al., 2019; Chen et al., 2020). In addition, Nanopore platforms represent useful tools for clinical application, owing to their real-time analysis and long reads for antibiotic resistance prediction (Chiu and Miller, 2019; Leggett et al., 2020). Compared with traditional clinical methods, Nanopore sequencing, as used in our study, requires less than 1 h for data analysis and a turnaround time (TAT) of approximately 6–8 h to identify pathogens in clinical samples. In this study, Nanopore sequencing was used to detect *T. whipplei* in the bronchoalveolar lavage fluid (BALF) of three patients with interstitial lung disease (ILD). To our knowledge, this is the first report of *T. whipplei* identification using Nanopore sequencing.

## Materials and Methods

### Sample Collection

On January 28, 2021, we began performing Nanopore sequencing to identify the pathogens in BALF or sputum samples collected from 418 patients, according to the diagnostic criteria for community-acquired pneumonia (Metlay et al., 2019), hospital-acquired and ventilator-associated pneumonia (Kalil et al., 2016) and were suspected to have pulmonary infection that was diagnosed by professional clinicians. The inclusion criteria were symptoms, such as fever, cough, expectoration, dyspnea, and abnormal imaging findings, such as pulmonary shadows, space-occupying lesions, and other signs of pulmonary infection. Demographic information, laboratory data, clinical symptoms, imaging examination results, diagnosis, and treatment history were recorded. This study was approved by the Peking University People’s Hospital Institutional Review Board (No. 2019PHB010-01). All samples were obtained with the consent of the patients.

### DNA Extraction, Nanopore Sequencing, and Real-Time Polymerase Chain Reaction

DNA was extracted from BALF or sputum samples using Quick-DNA/RNA™ Viral Kit (Zymo Research, Irvine, CA, United States). Human DNA was removed using saponin, as described previously (Charalampous et al., 2019). Sterile deionized water was used as a negative control. DNA concentrations were determined using the Qubit dsDNA HS Assay Kit (Thermo Fisher Scientific, Waltham, MA, United States). Oxford Nanopore Technology (ONT, Oxford, United Kingdom) libraries were prepared using a PCR barcoding kit (SQK-PBK004). Sequencing was performed using an ONT instrument (GridION X5). Clean reads were obtained after short reads (length ≤ 500 bp) and low-quality reads (mean *q*-score ≤ 8) removal. Subsequently, reads for the host DNA were removed by aligning to the human reference genome (GRCh38) using minimap2 (Version 2.14-r883). The remaining reads were mapped using the Centrifuge software (version 1.0.4) for taxonomic classification. The sequenced data were submitted to the Short Read Archive under the Bioproject PRJNA754208. After the sequencing and analysis were completed, a Pubmed search was conducted to determine whether the detected species cause pneumonia. The positive pathogens were defined as those with unique reads>3 (for bacteria) or>1 (for fungi) (Gu et al., 2021). *T. whipplei*-specific PCR primers were TW27 forward (5′-TGTTTTGTACTGCTTGTAACAGGATCT-3′) and TW182 reverse (5′-TCCTGCTCTATCCCTCCTATCATC-3′). The TaqMan probe (27 forward–182 reverse, 6-FAM-AGAGATACATTTGTGTTAGTTGTTACA-TAMRA) was added to the reaction mix. If the result was positive, a second PCR was performed to confirm the result using another set of primers [TW13 forward (5′-TGAGTGATGGTATGTCTGAGAGATATGT-3′) and TW163 reverse (5′ –TCCATAACAAAGACAACAACCAATC-3′)] and a different TaqMan probe (13 forward–163 reverse, 6-FAM-AGAAGAAGATGTTACGGGTTG-TAMRA). Quantitative PCR (qPCR) was performed using an Applied Biosystems 7500 system (Thermo Fisher Scientific), as described previously (Fenollar et al., 2008).

## Results

Among the patients for whom Nanopore sequencing was conducted, *T. whipplei* was detected in BALF samples of three patients (3/418, 0.72%), diagnosed with interstitial pneumonia by their clinicians, based on the laboratory rules and opinion of a clinical specialist. The detailed medical records of each patient are described below.

### Case Descriptions

**FIGURE 1:**
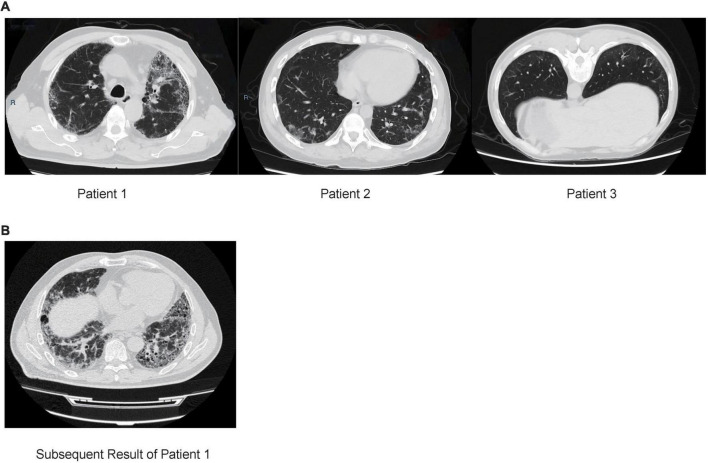
**(A)** Chest computed tomography (CT) of three patients with interstitial lung disease. Patient 1: CT results of the lower lobes of the lungs under the pleura are shown. Patients 2: CT result showing multiple grid shadows in both lungs. Patient 3: CT results showing subpleural mesh shadow and ground-glass opacity of both lungs. **(B)** CT result of patient 1 after 5 months, multiple ground-glass patchy shadows and grid honeycomb shadows in both lungs.

Patient 1 (male; age, 64) was from the Hebei Province, China and was admitted to the hospital due to a 4-year history of cough and dyspnea for 6 months (without fever, expectoration, and hemoptysis). Chest computed tomography (CT) showed that the lower lobes of the lungs were shadowed under the pleura, which was consistent with the characteristics of pulmonary fibrosis (Figure 1A). Routine blood tests, procalcitonin, and erythrocyte sedimentation rate (ESR) were normal. C-reactive protein (CRP) level was slightly higher (8.9 mg/L, normal: 0.068–8.2 mg/L). Etiology analysis, which included cell culture testing and diagnostic tests for tuberculosis, *Mycoplasma pneumoniae* and human cytomegalovirus infection, yielded negative results. Percentages of neutrophils (25.5%) and eosinophils (11.5%) were higher in BALF samples. Presence of carbon foam deposition in lung interstitial fibrous tissue was confirmed via transbronchial lung biopsy (TBLB) with positive periodic acid-Schiff (PAS) staining. The patient was negative for autoantibodies. To determine the cause of the disease, we performed Nanopore sequencing, with *T. whipplei* being the only pathogen identified. These results were subsequently verified via qPCR using two pairs of primers (Table 1). While admitted in the hospital, the patient was treated without any antibiotics, and pirfenidone was prescribed for treating pulmonary fibrosis after discharge. After 5 months, the patient still had dyspnea and was treated with cephalosporins; the result of subsequent CT also revealed ILD (Figure 1B). As suggested by a professional clinician, he was treated with streptomycin (1 g per day) together with penicillin G (1.2 million U per day) for 2 weeks and then with trimethoprim–sulfamethoxazole (0.96 g, twice daily), following which dyspnea was improved significantly.

**TABLE 1:** Summary of metagenomic sequencing and qPCR results of patients.

Patient no./sex/age, year	Final diagnosis	Pathogens	Reads (no.)	Primer 1 (Ct)	Primer 2 (Ct)
Patient 1/Male/64	IPF; Type 2 diabetes	*Tropheryma whipplei*	1,385	33.26	36.29
Patient 2/Male/31	Dermatomyositis; ILD	*Tropheryma whipplei*	836	31.68	32.01
Patient 3/Male/59	ILD	*Tropheryma whipplei*	285	28.82	28.80

*Ct, Cycle threshold; IPF, idiopathic pulmonary fibrosis; ILD, interstitial lung disease.*

Patient 2 (male; age: 31), from the Shandong Province, China, was immunosuppressed because of undergoing 1-year treatment for dermatomyositis and presented with fever, cough, intermittent diarrhea, weight loss, and arthralgia. Chest CT results showed multiple grid shadows in both lungs, indicating the presence of interstitial pneumonia (Figure 1A). White blood cell count, CRP level, and platelet count were normal, and the hemoglobin level was low (112 g/L, normal: 120–165 g/L). The patient was negative for autoantibodies. As per etiology analysis, the patient was negative for *Legionella pneumophila*, *Mycoplasma pneumoniae*, *Chlamydia pneumoniae*, *Rickettsia*, adenovirus, respiratory syncytial virus, influenza A/B viruses, parainfluenza virus, human alphaherpesvirus 1, rubella virus, *Toxoplasma gondii*, *Cryptococcus*, and *Pneumocystis jiroveci*, but positive for human cytomegalovirus and galactomannan in BALF. Nanopore sequencing results confirmed that *T. whipplei* (reads: 836) was the most abundant species, followed by *Streptococcus mitis* (reads: 130) and *Candida albicans* (reads: 6). The positive results for *T. whipplei* were confirmed by qPCR (Table 1). The patient was treated with voriconazole (200 mg, q12h), levofloxacin (400 mg, qd), ganciclovir (150 mg, qd), and sulfamethoxazole (2 pills, tid). The patient was discharged upon recovering from the rash, and was without a medical record as of date.

Patient 3 (male; age: 59), from the Shandong Province, China, had a dry cough when smelling a pungent odor or cold air every year. Chest CT revealed the presence of a subpleural mesh shadow and ground-glass opacity in both lungs, which led to an interstitial pneumonia diagnosis (Figure 1A). White blood cell count and ESR were normal. Given the negative results, additional tests were performed to assess the etiology of the symptoms, which included culture analysis and testing for presence of *Mycoplasma pneumoniae*, *Chlamydia pneumoniae* infection, galactomannan, and tuberculosis in BALF samples. In addition, the patient was positive (1:100) for anti-endothelial cell antibodies, representing a risk for the development of connective tissue disease in the future. Percentages of neutrophils, eosinophils, macrophages, and lymphocytes were 3.5, 1.5, 88, and 7%, respectively. Positive results were obtained using Masson’s trichrome staining of TBLB specimens. Nanopore sequencing was performed to investigate the cause of the disease; *T. whipplei* was the most abundant species (reads: 285), followed by *Pseudomonas poae* (reads: 15) and *Streptococcus pseudopneumoniae* (reads: 8). These results were subsequently confirmed via qPCR using two pairs of primers (Table 1). The patient was treated without antibiotics and was discharged when the sequencing results were obtained; no further medical record was obtained to this day.

## Discussion

Although WD was first described in 1907, *T. whipplei* was only successfully cultured after a century (Raoult et al., 2000), which improved scientific knowledge on the infections caused by this bacterium. For classic WD, common symptoms include gastrointestinal, joint, neurologic, and cardiac involvement. Fenollar et al. (2007) reported that 87% of WD patients are male, with 93, 81, and 73% of cases presenting weight loss, diarrhea, and arthralgia, respectively. Lung involvement is an unusual but classic manifestation of WD (Lagier et al., 2017), and *T. whipplei* infection can occur in patients with aspiration pneumonia, ventilator-associated pneumonia, and community-acquired pneumonia (Bousbia et al., 2010). The prevalence of *T. whipplei* in our study was lower than that reported by Lagier et al. (2016) (88/1438, 6.1%), based on a qPCR method. Bousbia et al. (2010) demonstrated a *T. whipplei* prevalence of 3% (6/210) in BALF samples collected from intensive care units employing 16S rDNA and specific quantitative PCR. The reason for the lower prevalence of *T. whipplei* in our study might be the higher sensitivity of specific quantitative PCR than that of Nanopore sequencing. However, in a large metagenomic study, Morris et al. (2013) reported that the prevalence of *T. whipplei* was surprisingly 26% in healthy subjects. Zhang and Xu (2021) showed that *T. whipplei* can affect the pulmonary parenchyma. In our study, *T. whipplei* was detected in three patients with ILD, which was consistent with the findings of Urbanski et al. (2012). All patients had respiratory symptoms with cough, and only one patient had fever. Patient 2 was immunosuppressed due to previous dermatomyositis treatment and had intermittent diarrhea, weight loss, and arthralgia, which are risk factors for *T. whipplei* infection (Marth and Raoult, 2003). It remains difficult to gauge the effect of *T. whipplei* in the respiratory system, and the Koch’s assumptions should be proved using an animal model in the future.

One strategy to diagnose WD is to use the results of PAS staining of small-bowel biopsy specimens, in addition to PCR assays (Fenollar et al., 2007). However, another study reported histologic examination of small bowel biopsy specimens as the first step, followed by PCR if the histologic findings are negative (Olmos et al., 2006). In this study, we performed Nanopore sequencing to identify pathogens in BALF samples, and the results were subsequently confirmed via qPCR using two paired primers (Table 1). The PCR products were further identified via Sanger sequencing that all results were mapped to *T. whipplei* with BLAST (Table 2). *T. whipplei* was identified as the only pathogen in patient 1, with positive PAS staining in BALF samples. Due to the continual dyspnea and the CT result of ILD, he is undergoing anti-*T. whipplei* treatment as suggested by a clinician after 5 months and has shown significant improvement. In the other two patients, *T. whipplei* was the most abundant organism, as detected by Nanopore sequencing, and most of the other identified species were normal flora of the respiratory tract. However, they were not treated for *T. whipplei* and were without subsequent medical records. Therefore, the effect of *T. whipplei* in pulmonary infection should be considered comprehensively. All Nanopore results were generated within 1 h, owing to the real-time analysis pipeline. Hence, in this study, we demonstrate that Nanopore-based sequencing can be a powerful tool to detect rare pathogens with a short TAT, which could improve clinical diagnosis.

**TABLE 2:** The Sanger sequencing result of qPCR product with *T. whipplei* for each patients.

Patients no.	Sanger sequencing result (Primer 1)	Sanger sequencing result (Primer 2)
1	TTGTTTTGTACTGCTTGTAACAGGATCTATTAGGAGAGATACATTTGTGTTAGT TGTTACACATACTTCTTGTGTATTTGTATTACTTACTCTTGTGTATTTGGTATT AGATGAAACAGATGTAGATGAAACAGATGATAGGAGGGATAGAGCAGG AACCT	TGAGTGATGGTATGTCTGAGAGATATGTGTTATCTATCTGTTTGTGTATGGGAA CAGTTATCTTTGGTTTCTCTGTTACATGTATGTCAAAGAAGAAGATGTTACG GGTTGTTAGGAGAGCTATGAGATTGGTTGTTGTCTTTGTTATGGA
2	TGTTTTGTACTGCTTGTAACAGGATCTATTAGGAGAGATACATTTGTGTTA GTTGTTACACATACTTCTTGTGTATTTGTATTACTTACTCTTGTGTATTTGGT ATTAGATGAAACAGATGTAGATGAAACAGATGATAGGAGGGATAGAG CAGGA	TTGAGTGATGGTATGTCTGAGAGATATGTGTTATCTATCTGTTTGTGTATGG GAACAGTTATCTTTGGTTTCTCTGTTACATGTATGTCAAAGAAGAAGATGTT ACGGGTTGTTAGGAGAGCTATGAGATTGGTTGTTGTCTTTGTTATGGAA
3	TTGTTTTGTACTGCTTGTAACAGGATCTATTAGGAGAGATACATTTGTGTTA GTTGTTACACATACTTCTTGTGTATTTGTATTACTTACTCTTGTGTATTTGGT ATTAGATGAAACAGATGTAGATGAAACAGATGATAGGAGGGATA GAGCAGGAA	GTTTGAGTGATGGTATGTCTGAGAGATATGTGTTATCTATCTGTTTGTGTATGG GAACAGTTATCTTTGGTTTCTCTGTTACATGTATGTCAAAGAAGAAGATGTT ACGGGTTGTTAGGAGAGCTATGAGATTGGTTGTTGTCTTTGTTATGGAA

There were some limitations to this study. First, the PAS staining or histologic examination of small-bowel biopsy specimens, which is the gold standard for WD, was not performed due to short hospital stay of the patients. Second, the patients were not treated with antibiotics immediately in hospital due to our retrospective study.

In summary, this is the first report of *T. whipplei* being detected using Nanopore sequencing of BALF samples, which suggests that this analytical approach can be used to improve the diagnosis of infectious diseases caused by difficult-to-culture pathogens. Moreover, these cases highlight that it is essential to consider the possibility of *T. whipplei* infection as a diagnosis in middle-aged men with ILD of unknown cause, and the clinician should combine other clinical laboratory findings such as PAS staining to decide the treatment for *T. whipplei*.

## Data Availability Statement

The datasets presented in this study can be found in online repositories. The names of the repository/repositories and accession number(s) can be found below: https://dataview.ncbi.nlm.nih.gov/object/PRJNA754208.

## Ethics Statement

This study was approved by the Peking University People’s Hospital Institutional Review Board (No. 2019PHB010-01). All samples were obtained with the consent of the patients.

## Author Contributions

HW conceived, designed, supervised the study, revised the draft of the manuscript, and attests that all listed authors meet the authorship criteria and that no others meeting the criteria have been omitted. YG acquired the data and wrote the draft of the manuscript. YG and ZL analyzed and interpreted the data. YG, LS, and LL conducted the clinical work associated with the study. ZL provided technical support. All authors approved the final version of the manuscript.

## Conflict of Interest

ZL was employed by Simcere Diagnostics Co., Ltd. and Nanjing Simcere Medical Laboratory Science Co., Ltd. The remaining authors declare that the research was conducted in the absence of any commercial or financial relationships that could be construed as a potential conflict of interest.

## Publisher’s Note

All claims expressed in this article are solely those of the authors and do not necessarily represent those of their affiliated organizations, or those of the publisher, the editors and the reviewers. Any product that may be evaluated in this article, or claim that may be made by its manufacturer, is not guaranteed or endorsed by the publisher.
